# AI-Powered Renal Diet Support: Performance of ChatGPT, Bard AI, and Bing Chat

**DOI:** 10.3390/clinpract13050104

**Published:** 2023-09-26

**Authors:** Ahmad Qarajeh, Supawit Tangpanithandee, Charat Thongprayoon, Supawadee Suppadungsuk, Pajaree Krisanapan, Noppawit Aiumtrakul, Oscar A. Garcia Valencia, Jing Miao, Fawad Qureshi, Wisit Cheungpasitporn

**Affiliations:** 1 Division of Nephrology and Hypertension, Department of Medicine, Mayo Clinic, Rochester, MN 55905, USA; ahmad_qarajeh99@hotmail.com (A.Q.); charat.thongprayoon@gmail.com (C.T.); s.suppadungsuk@hotmail.com (S.S.); pajaree_fai@hotmail.com (P.K.); garciavalencia.oscar@mayo.edu (O.A.G.V.); miao.jing@mayo.edu (J.M.); qureshi.fawad@mayo.edu (F.Q.); 2 Faculty of Medicine, University of Jordan, Amman 11942, Jordan; 3 Chakri Naruebodindra Medical Institute, Faculty of Medicine Ramathibodi Hospital, Mahidol University, Samut Prakan 10540, Thailand; 4 Department of Internal Medicine, Faculty of Medicine, Thammasat University, Pathum Thani 12120, Thailand; 5 Department of Medicine, John A. Burns School of Medicine, University of Hawaii, Honolulu, HI 96813, USA; noppawit@hawaii.edu

**Keywords:** chronic kidney disease, CKD, renal diets, hyperkalemia, hyperphosphatemia, AI technology, ChatGPT, Bard AI, Bing Chat, potassium content, phosphorus content, food assessment, healthcare providers, efficacy evaluation, dietary planning

## Abstract

Patients with chronic kidney disease (CKD) necessitate specialized renal diets to prevent complications such as hyperkalemia and hyperphosphatemia. A comprehensive assessment of food components is pivotal, yet burdensome for healthcare providers. With evolving artificial intelligence (AI) technology, models such as ChatGPT, Bard AI, and Bing Chat can be instrumental in educating patients and assisting professionals. To gauge the efficacy of different AI models in discerning potassium and phosphorus content in foods, four AI models—ChatGPT 3.5, ChatGPT 4, Bard AI, and Bing Chat—were evaluated. A total of 240 food items, curated from the *Mayo Clinic Renal Diet Handbook* for CKD patients, were input into each model. These items were characterized by their potassium (149 items) and phosphorus (91 items) content. Each model was tasked to categorize the items into high or low potassium and high phosphorus content. The results were juxtaposed with the *Mayo Clinic Renal Diet Handbook’s* recommendations. The concordance between repeated sessions was also evaluated to assess model consistency. Among the models tested, ChatGPT 4 displayed superior performance in identifying potassium content, correctly classifying 81% of the foods. It accurately discerned 60% of low potassium and 99% of high potassium foods. In comparison, ChatGPT 3.5 exhibited a 66% accuracy rate. Bard AI and Bing Chat models had an accuracy rate of 79% and 81%, respectively. Regarding phosphorus content, Bard AI stood out with a flawless 100% accuracy rate. ChatGPT 3.5 and Bing Chat recognized 85% and 89% of the high phosphorus foods correctly, while ChatGPT 4 registered a 77% accuracy rate. Emerging AI models manifest a diverse range of accuracy in discerning potassium and phosphorus content in foods suitable for CKD patients. ChatGPT 4, in particular, showed a marked improvement over its predecessor, especially in detecting potassium content. The Bard AI model exhibited exceptional precision for phosphorus identification. This study underscores the potential of AI models as efficient tools in renal dietary planning, though refinements are warranted for optimal utility.

## 1. Introduction

Chronic kidney disease (CKD), a condition characterized by the gradual decline in kidney function over time, poses various challenges for patients and healthcare providers [1]. According to the World Health Organization (WHO) and multiple studies on population health, CKD affects around 13% of people worldwide [2]. This means that hundreds of millions of individuals are dealing with this condition, making it a significant global issue. Additionally, as the global population ages and conditions such as diabetes and hypertension become more prevalent (which are risk factors for CKD), the prevalence of CKD is expected to increase further [3,4]. One concerning aspect of CKD is its connection to imbalances in potassium and phosphorus metabolism. The kidneys play a role in maintaining the proper levels of these minerals by filtering out excess amounts from the blood and excreting them through urine. However, when kidney function is compromised, as seen in CKD, their ability to maintain this balance diminishes and leads to an imbalance in the levels of potassium (hyperkalemia) and phosphorus (hyperphosphatemia) in the bloodstream [5]. These conditions can have implications for cardiovascular health and musculoskeletal wellbeing, necessitating close monitoring of dietary intake and implementing interventions as necessary [6,7].

The management of these complications heavily relies on a specialized renal diet tailored for CKD patients. This diet entails the meticulous selection and consumption of foods based on their potassium and phosphorus content [8]. However, this task is far from straightforward. The nutritional composition of foods can vary significantly, and even minor deviations from recommended intake levels can lead to severe health implications. Healthcare providers are faced with the demanding task of thoroughly assessing the nutritional components of foods. This task is not only laborious but also time-intensive, presenting additional hurdles for healthcare providers [9].

In the context of the rapidly evolving technological landscape and its integration into healthcare, potential solutions to this predicament have surfaced. We currently find ourselves in the digital age, where artificial intelligence (AI) has transitioned from a futuristic notion to a contemporary reality. While the integration of AI into various sectors such as finance and transportation has been well-documented, its potential within the realm of healthcare is arguably the most revolutionary [10,11]. AI models such as ChatGPT, Bard AI, and Bing Chat transcend being mere algorithms: they epitomize the culmination of human inventiveness and technological advancement. These models possess the capacity to analyze vast volumes of data with remarkable precision, learn intricate patterns, and yield consistent outcomes [12]. The conceivable applications of such AI models in the context of CKD and CKD dietary planning are manifold [13,14]. Initially, they can serve as educative tools, elucidating the complexities of renal diets for patients. Furthermore, these models can function as dependable supplements for healthcare professionals, streamlining the laborious process of dietary assessment. However, prior to the integration of these models into clinical practice, it is imperative to ascertain their effectiveness, accuracy, and reliability.

The potential applications of AI models in the area of CKD dietary planning are multifaceted. Primarily, they serve as educational instruments, unraveling the intricacies of renal diets for patients. Secondly, healthcare practitioners can rely on these models as valuable adjuncts, simplifying the intricate task of dietary evaluation. Nevertheless, before incorporating these models into practical healthcare settings, a thorough assessment of their effectiveness, precision, and dependability is imperative. The advent of the AI era has brought about the development of models such as ChatGPT, Bard AI, and Bing Chat.

Generative AI models have gained prominence in recent years due to their ability to generate new content by learning patterns and structures from vast amounts of data [15,16,17,18]. These models are designed to understand context, predict subsequent sequences, and produce information that is coherent and contextually relevant. Such attributes make them potential tools in diverse applications, including healthcare. ChatGPT 3.5 and ChatGPT 4 are both products of OpenAI, with the latter being an advanced version of the former. ChatGPT 4 boasts improved performance, finer-tuned algorithms, and an enhanced ability to handle complex tasks over its predecessor—ChatGPT 3.5 [15,16,17,18,19,20]. Both models are designed for a myriad of tasks, from straightforward information retrieval to complex problem solving. Bard AI is particularly strong in comprehending and generating narratives. It can understand the context of a story and create new plot points, characters, and dialogue that are consistent with the overall narrative. Bing Chat, developed by Microsoft, has been optimized for web-based interactions and tends to generate concise and direct responses [15,16,17,18]. This model, with its swift processing capabilities, could be particularly beneficial in scenarios where rapid information retrieval is essential.

These AI models, equipped with extensive repositories of information and advanced algorithms, possess the capability to aid in intricate tasks, including dietary analysis. Beyond serving as mere tools, these models can fulfill a dual role: enlightening patients about their dietary requirements and furnishing healthcare professionals with a trustworthy resource for food assessment. The rationale behind evaluating these specific models was due to their prominence in the AI community and their potential applicability to the healthcare sector. While all these models operate on the foundation of generative AI, they each have unique strengths, algorithms, and operational nuances that we believed would bring varied perspectives and capabilities to the intricate task of renal diet assessment.

To assess the effectiveness of different AI models in accurately determining the potassium and phosphorus content in foods, this study evaluated four AI models—ChatGPT 3.5, ChatGPT 4, Bard AI, and Bing Chat.

## 2. Materials and Methods

### 2.1. Materials and Procedures

The core objective of this study was to assess the efficacy of diverse AI models in accurately determining the potassium and phosphorus content of dietary items, which is a pivotal consideration for individuals adhering to a renal diet. The AI models under investigation encompassed ChatGPT 3.5, ChatGPT 4, Bard AI, and Bing Chat.

### 2.2. Selection and Compilation of Dietary Items

**Figure 1:**
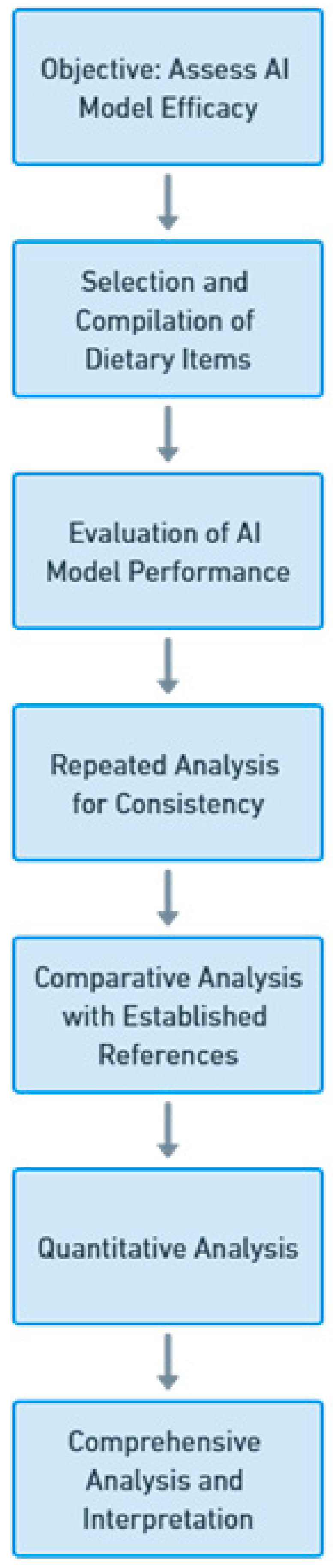
Flowchart overview of this study.

To execute this study, we meticulously compiled a comprehensive assortment of 240 dietary items, which were meticulously sourced from the reputable Mayo Clinic’s renal diet compendium. This compendium is renowned as a trustworthy reference for individuals grappling with CKD and its dietary management. The selection process encompassed 149 dietary items that were characterized by their potassium content, while an additional 91 items were categorized by their phosphorus content. The assortment of these dietary items reflects a diverse spectrum of choices frequently encountered within a renal diet regimen (Figure 1).

### 2.3. Evaluation of AI Model Performance

Each of the chosen AI models was tasked with the responsibility of categorizing the curated dietary items based on their potassium and phosphorus content. This categorization procedure involved classifying the items into distinct categories: those possessing high or low potassium content, as well as those with high phosphorus content. For the purpose of generating responses from the AI models, the following prompts were utilized:

1. Is ___ considered a low or high potassium/phosphorus diet?
2. Classify the following as low or high potassium/phosphorus diet: ___.

Furthermore, each AI model was tasked with categorizing the selected dietary items based on their potassium and phosphorus content on their respective dates as follows:

ChatGPT 3.5: first session on 10th of April and second session on 24 April 2023.

ChatGPT 4: 10 August 2023.

Bard AI: 15 August 2023.

Bing Chat: 13 August 2023.

### 2.4. Repeated Analysis for Ensured Consistency

In order to mitigate the likelihood of fortuitous results and to affirm the consistent analytical capabilities of the AI models, the entire methodology was repeated twice, separated by a two-week interval between each instance. This procedural iteration aimed to account for potential variations and temporal fluctuations in the performance of the AI models.

The interval of two weeks between each instance was a deliberate choice made after considering multiple factors. One primary consideration was the evolving nature of AI models, which undergo frequent updates. A two-week span minimizes the risk of significant model alterations, ensuring consistency in our evaluations. Additionally, from an operational standpoint, this duration allowed for comprehensive assessment, adjustments, and preparations for the subsequent tests. It also accounted for potential short-term fluctuations in AI responses due to transient technical factors. Thus, this time frame represented a balanced approach that prioritized both methodological rigor and feasibility.

### 2.5. Comparative Analysis with Established References

The outcomes yielded by the AI models were subsequently juxtaposed with the dietary recommendations furnished in the *Mayo Clinic Renal Diet Handbook*. This comparison enabled the scrutiny of the accuracy and correspondence of the AI-generated classifications with the well-regarded standards advocated by an authoritative source.

To determine the cut-offs for low vs. high potassium and phosphorus content in dietary items, we relied on standard guidelines and inputs from dietitians at the Mayo Clinic, along with established recommendations from several renowned organizations such as the National Kidney Foundation [21,22]. For potassium, the criteria are as follows:

The Academy of Nutrition and Dietetics suggests a limitation of potassium to 2–3 g per day for patients on dialysis or with end-stage renal disease. Translated, this is equivalent to 2000–3000 mg of potassium daily. Given standard serving sizes, this guideline aligns with the recommendations to restrict high potassium foods (200–400 mg per serving) to 1–2 servings daily [23].

The National Kidney Foundation proposes a daily intake of 1500–2700 mg of potassium for patients with varying severities of chronic kidney disease. Again, the lower end of this spectrum emphasizes restricting high potassium foods to 1–2 servings every day [21].

The FDA stipulates that foods comprising more than 200 mg of potassium per serving are viewed as high in potassium [22].

For phosphorus, the criteria are as follows:

High phosphorus foods exceeding 300 mg of phosphorus per serving or surpassing 30% daily value (DV) for phosphorus. DV for phosphorus stands at 1250 mg per day. Consequently, 10% DV corresponds to 125 mg phosphorus per serving. It is pertinent to note that phosphorus levels can vary extensively depending on the food type, brand, and preparation method. Thus, inspecting ingredient labels for phosphorus additives is also advocated [24].

### 2.6. Quantitative Analysis

To encapsulate the performance of the AI models in categorizing the dietary items, we employed descriptive statistical techniques. Specifically, percentages and frequencies were computed to quantify the accuracy of the categorizations for both high and low potassium content, as well as high phosphorus content. Moreover, we conducted rigorous statistical analyses to gauge the degree of concordance between the outcomes produced by the AI models and the stipulations outlined in the *Mayo Clinic Renal Diet Handbook*. Notably, methodologies such as Cohen’s kappa coefficient were employed to measure the level of agreement between the different entities involved.

### 2.7. Comprehensive Analysis and Interpretation

The results derived from the AI model categorizations were subjected to meticulous analysis and comprehensive interpretation. An exhaustive evaluation of the precision of each AI model in correctly classifying the dietary items based on their potassium and phosphorus content was conducted. Furthermore, an assessment of the degree of concurrence between the AI models and the recommendations in the *Mayo Clinic Renal Diet Handbook* was undertaken. The insights garnered from this analytical endeavor shed light on the effectiveness of the AI models in guiding individuals afflicted with CKD towards judicious dietary decisions.

### 2.8. AI Algorithm Strategy and Dataset Value Adherence

The selected AI models, namely ChatGPT 3.5, ChatGPT 4, Bard AI, and Bing Chat, utilize large-scale transformer architectures, which are adept at pattern recognition derived from extensive training datasets. These models were oriented to the context of renal diets through specific priming prompts. As dietary items were fed into the models, the algorithms matched the input with recognized patterns from their training, predicting the most probable categorization for potassium and phosphorus content. It is vital to recognize that these models, while extensive in their knowledge base, operate based on probabilistic pattern matching rather than human-like understanding.

## 3. Results

**Figure 2:**
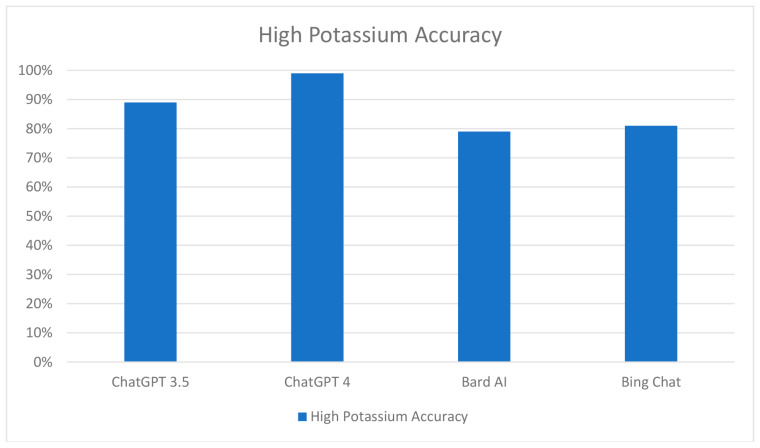
Performance comparison of chatbots in the classification of food items with high potassium content.

In this research, the following four AI models were tested: ChatGPT 3.5, ChatGPT 4, Bard AI, and Bing Chat. The results revealed that ChatGPT 3.5 accurately identified 66% of the food items (98 out of 149) as either high or low in potassium. Specifically, it correctly categorized 38% (26 out of 68) of foods as low in potassium and 89% (72 out of 81) of foods as high in potassium (Supplementary Table S1). The concordance between two separate ChatGPT 3.5 sessions was 81% (121 out of 149) for foods containing potassium, with a 79% (54 out of 68) agreement for low potassium items and an 83% (67 out of 81) agreement for high potassium items. ChatGPT 4 outperformed its predecessor, correctly identifying 81% (121 out of 149) of food items. It accurately classified 60% (41 out of 68) of items as low in potassium and achieved an accuracy of 99% (80 out of 81) for high potassium items (Figure 2).

**Figure 3:**
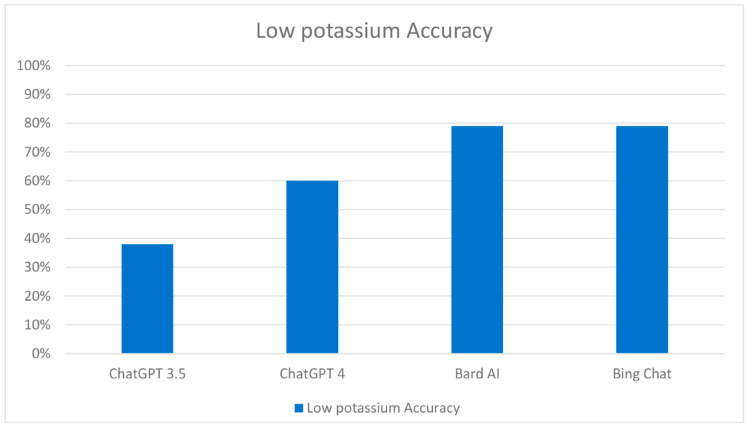
Performance comparison of chatbots in the classification of food items with low potassium content.

The Bard AI model accurately identified 79% (118 out of 149) of food items. This included a 79% (54 out of 68) accuracy for low potassium items and an identical 79% (64 out of 81) accuracy for high potassium items. Bing Chat demonstrated similar results, correctly categorizing 81% (120 out of 149) of food items. It achieved 79% (54 out of 68) accuracy for low potassium items and 81% (66 out of 81) accuracy for high potassium items (Figure 3).

In the high potassium diet category, all models, including GPT 3.5’s first and second tests, GPT 4, Bard, and Bing, demonstrate consistently correct results for a wide range of foods, such as acorn squash, apricots, baked beans, bananas, and more. However, slight variations in performance are observed for certain foods. For instance, for some foods such as chocolate milk and elderberries, there are instances of incorrect identifications by specific AI models. On the other hand, in the low potassium diet category, the accuracy of the AI models’ predictions is more mixed. While some foods are accurately identified across the board, there are instances where models, such as GPT 3.5 and Bing, make incorrect predictions. It is worth noting that even within this category certain AI models consistently provide correct answers for foods such as raspberry, iceberg lettuce, and others. However, there are foods such as avocado, sour cherries, and some variations of fruit juices where multiple models exhibit inaccuracies.

**Figure 4:**
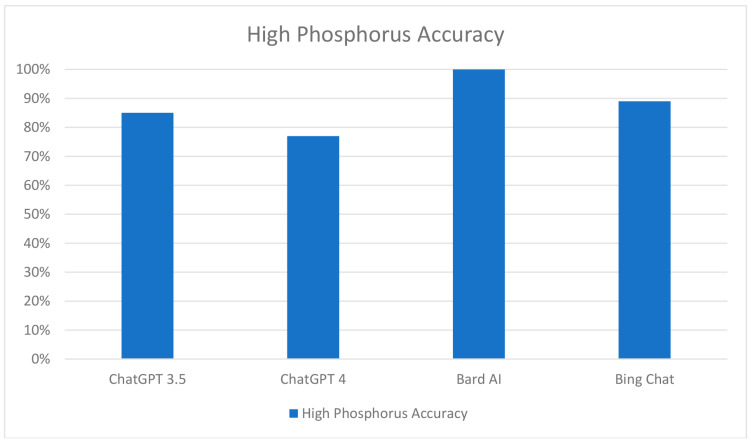
Performance comparison of chatbots in the classification of food items with high phosphorus content.

In terms of high phosphorus diets, the AI models displayed varying accuracy levels. ChatGPT 3.5 identified 85% (77 out of 91) of food items as high in phosphorus (Supplementary Table S2). The concordance between two ChatGPT sessions was 90% (82 out of 91) for foods containing phosphorus. ChatGPT 4 accurately classified 77% (70 out of 91) of food items with high phosphorus content. The Bard AI model demonstrated strong performance by correctly identifying all 91 food items as high in phosphorus (Figure 4). Similarly, Bing Chat accurately classified 89% (81 out of 91) of food items as high in phosphorus.

Across a range of foods, including different types of cheeses, dairy products, legumes, and some beverages, the models exhibit good accuracy in their predictions. Foods such as blue cheese, cheddar cheese, kidney beans, black beans, and peas are correctly identified by all models. However, there are instances of discrepancies in the models’ predictions. For example, with ricotta cheese, cheese spread, hummus, lentils, and firm tofu, some AI models make incorrect identifications. Additionally, there are cases where the AI models have inconsistent results among themselves, such as in the identification of éclairs, chocolate cream pie, and coconut cream pie.

## 4. Discussion

This study explored the capabilities of four well-known artificial intelligence models, comprising ChatGPT 3.5, ChatGPT 4, Bard AI, and Bing Chat, revealing insights into their potential to enhance medical nutrition therapy. As AI-driven assistants become increasingly prevalent, understanding their strengths and limitations in categorizing nutrient content is crucial for their responsible integration into clinical care. This study’s primary focus was to assess their accuracy in classifying potassium and phosphorus levels, which bear significant implications for certain medical conditions.

Together, the results underscore the progressing expertise of expansive language models in precisely classifying nutritional content present in various foods. The latest iterations, namely ChatGPT 4 and Bing Chat, showcased the highest accuracy rates, exceeding 80%, in effectively categorizing potassium content. Similarly, Bard AI and Bing Chat exhibited strong performance by accurately categorizing 89–100% of high phosphorus foods. These outcomes suggest that AI possesses the potential to enhance nutrition education and counseling, particularly in cases where potassium and phosphorus restriction is vital. However, inconsistencies across the models remain, underscoring the necessity for caution and human oversight when employing AI for nutritional guidance in medical contexts. Despite the notable progress seen in models such as ChatGPT 4 and Bing Chat, errors still occur in at least one out of every five foods or more. Furthermore, some models seem better equipped to categorize high potassium foods as opposed to low potassium ones. Hasty integration of AI into nutrition care could potentially lead to harm through inappropriate recommendations.

This issue is especially notable for individuals diagnosed with CKD, a condition that impacts a considerable proportion of the adult population in the United States [25]. Patients with declining kidney function face challenges in excreting potassium, thereby increasing the risk of fatal cardiac arrhythmias due to hyperkalemia [26]. In the context of CKD, where dietary potassium intake restriction is essential, the misclassification of high potassium foods as low potassium could jeopardize patients following potassium-restricted diets. The importance of phosphorus restriction is also evident in late-stage CKD patients, as it plays a role in preventing secondary hyperparathyroidism and cardiovascular disease [27,28]. Failure of an AI model to identify high phosphorus foods could disrupt effective phosphate management, which is particularly concerning considering the presence of hidden phosphorus additives in highly processed foods [29,30].

Although AI shows potential in categorizing potassium and phosphorus content to assist in nutritional counseling for conditions such as CKD, there remains a need for further enhancements to achieve accuracy levels of 90% or above. Moreover, ensuring transparency in an AI tool’s training methodology and validation testing is imperative for its secure integration into clinical practice [31,32,33]. Ideally, AI assistants should provide ranges of nutrient values rather than binary classifications of high/low potassium or phosphorus, contextualized within the patient’s clinical situation [34]. Potassium intake recommendations are highly individualized, contingent on the patient’s stage of CKD and treatment plan [35]. Similar personalization applies to phosphorus limits based on varying degrees of kidney function and individual factors [36]. AI’s role should involve tailoring nutrient recommendations to specific medical requirements, rather than employing one-size-fits-all categorizations.

This study’s findings suggest variations among AI models when applied to identical datasets, which could be influenced by the subjectivity inherent in determining the classification of nutrient content as “high” or “low”. To ensure safe application in medical settings, AI tools necessitate standardized datasets benchmarked against established clinical guidelines to prevent conflicting recommendations [37,38]. The observed variability between two ChatGPT 3.5 query sessions raises concerns about reliability. In clinical practice, consistent and aligned nutrition advice is essential for building patient trust [39]. Fluctuating recommendations from the same AI tool can lead to confusion and erode that trust. Addressing this issue of inconsistency will require enhanced training techniques as AI continues to mature.

It is crucial to emphasize that not all CKD patients necessitate a low potassium or low phosphorus diet. While our study highlights the importance of accurately categorizing nutrient content, it is essential to understand that dietary prescriptions for CKD patients are multifaceted and highly individualized. Various factors, including the stage of CKD, comorbid conditions, and individual patient needs, influence dietary recommendations [40,41]. Furthermore, numerous vegetables and fruits, while containing significant amounts of potassium, are packed with other essential nutrients and health benefits. Antioxidants, fibers, and other phytochemicals present in these foods play a crucial role in overall health [41]. Therefore, it is essential to strike a balance between restricting certain nutrients and ensuring the intake of other beneficial components. This nuance is vital for a comprehensive understanding of dietary recommendations in CKD and other medical conditions.

The potential implications of this study could have meaningful significance for individuals with CKD as well as healthcare professionals. As CKD management requires thorough attention to dietary potassium and phosphorus content, the integration of advanced AI models such as ChatGPT, Bard AI, and Bing Chat could transform the way renal diets are tailored and managed [42]. The findings from this study offer a glimpse into the potential impact of AI-powered solutions in addressing the complexities of renal nutrition.

**Figure 5:**
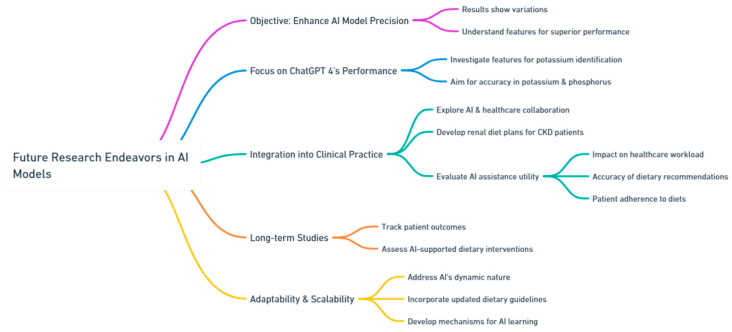
Future research endeavors within the domain of chatbot/AI models in renal diet.

Future research endeavors within this domain should focus on enhancing and fine-tuning the precision and dependability of AI models. While the results are promising, this study reveals variations in performance among the tested models. Further investigations could delve into the specific features and algorithms that contributed to ChatGPT 4’s superior performance in potassium identification. Understanding these factors could lead to the development of enhanced models that exhibit consistently high accuracy rates across both potassium and phosphorus categorizations. Additionally, future research might explore the integration of AI models into clinical practice. This could involve pilot programs where healthcare providers collaborate with AI systems to develop personalized renal diet plans for CKD patients. Such studies could assess the real-world utility of AI assistance, evaluating its impact on the workload of healthcare professionals, the accuracy of dietary recommendations, and patient adherence to prescribed diets. Long-term studies tracking patient outcomes could provide valuable insights into the effectiveness of AI-supported dietary interventions. Considering the dynamic nature of AI technology, future studies should also address the adaptability and scalability of these models. As medical knowledge evolves and dietary guidelines are updated, AI systems should be designed to seamlessly incorporate new information and recommendations. Research efforts could focus on developing mechanisms that allow AI models to learn and integrate the latest medical insights, ensuring that the dietary advice provided remains current and accurate (Figure 5).

Ethical deliberations play a critical role in the integration of AI within the realm of healthcare [43,44,45]. Future studies could delve into the ethical implications of AI-supported dietary counseling. Exploring topics such as patient autonomy, informed consent, and the role of human oversight in AI-generated recommendations could contribute to the establishment of ethical guidelines for integrating AI into medical nutrition therapy. Addressing these ethical concerns would be crucial for building trust between patients, healthcare providers, and AI systems [46]. As AI models become more integrated into healthcare settings, user experience and interaction design become increasingly important [47]. Future studies could explore how to optimize the user interface and experience of AI-powered dietary counseling tools. This might involve user surveys, focus groups, and usability testing to ensure that the AI tools are intuitive, user-friendly, and accessible to both healthcare professionals and patients.

It is important to acknowledge the limitations inherent in this study. Firstly, the evaluation of AI models was conducted within a controlled experimental environment using a specific dataset of food items from the *Mayo Clinic Renal Diet Handbook*. The real-world diversity of foods and variations in nutrient content may not have been fully represented, potentially impacting the models’ performance in practical clinical scenarios. Additionally, while ChatGPT, Bard AI, and Bing Chat exhibited varying degrees of accuracy, the reasons behind these discrepancies were not extensively explored. Future studies could delve into the underlying factors contributing to the models’ successes and limitations, offering a more comprehensive understanding of their functioning. Moreover, while the models were evaluated based on their ability to categorize foods into high or low potassium and phosphorus content, the nuances of recommended intake ranges for different stages of CKD were not fully considered. Tailoring dietary advice to the individual needs of CKD patients requires a more intricate understanding of their specific conditions and medical history. Lastly, this study primarily focused on the models’ accuracy in nutrient classification, leaving aside potential considerations such as user experience, usability, and the integration of AI recommendations into clinical workflows. These limitations underscore the need for ongoing research and refinement in the application of AI models to medical nutrition therapy.

## 5. Conclusions

This study provides an initial insight into how emerging AI models assess and classify nutrient content pertinent to medical nutrition therapy. While showing potential, the outcomes emphasize that AI still requires human oversight for independently recommending nutritional interventions. Nevertheless, AI’s potential to complement dietitians in crafting personalized meal plans is evident, provided that transparency, accuracy, consistency, and validation against clinical standards continue to improve. Instead of replacing healthcare professionals, AI is best positioned as a supplementary tool to enhance nutrition education and counseling while alleviating the workload on medical teams. As technology evolves, further investigation into the real-world implementation of AI for nutrition guidance is essential.

## Data Availability

Data supporting this study are available in the original publication, reports, and preprints that were cited in the reference citation.

## Supplementary Materials

The following supporting information can be downloaded at: https://www.mdpi.com/article/10.3390/clinpract13050104/s1. Table S1: Potassium Identification Results by Chatbots; Table S2: Accuracy of AI Models in Identifying High Phosphorus Foods.

## References

[1] Elendu C., Elendu R.C., Enyong J.M., Ibhiedu J.O., Ishola I.V., Egbunu E.O., Meribole E.S., Lawal S.O., Okenwa C.J., Okafor G.C. (2023). Comprehensive review of current management guidelines of chronic kidney disease. Medicine (Baltimore).

[2] Ammirati A.L. (2020). Chronic Kidney Disease. Rev. Da Assoc. Médica Bras..

[3] Ng J.K.C., Li P.K.T. (2018). Chronic kidney disease epidemic: How do we deal with it?. Nephrology.

[4] Bahrey D., Gebremedhn G., Mariye T., Girmay A., Aberhe W., Hika A., Teklay G., Tasew H., Zeru T., Gerensea H. (2019). Prevalence and associated factors of chronic kidney disease among adult hypertensive patients in Tigray teaching hospitals: A cross-sectional study. BMC Res. Notes.

[5] Hannedouche T., Fouque D., Joly D. (2018). [Metabolic complications in chronic kidney disease: Hyperphosphatemia, hyperkalemia and anemia]. Nephrol. Ther..

[6] Thongprayoon C., Kattah A.G., Mao M.A., Keddis M.T., Pattharanitima P., Vallabhajosyula S., Nissaisorakarn V., Erickson S.B., Dillon J.J., Garovic V.D. (2022). Distinct phenotypes of hospitalized patients with hyperkalemia by machine learning consensus clustering and associated mortality risks. Qjm.

[7] Thongprayoon C., Dumancas C.Y., Nissaisorakarn V., Keddis M.T., Kattah A.G., Pattharanitima P., Petnak T., Vallabhajosyula S., Garovic V.D., Mao M.A. (2021). Machine Learning Consensus Clustering Approach for Hospitalized Patients with Phosphate Derangements. J. Clin. Med..

[8] Hershey K. (2018). Renal Diet. Nurs. Clin. N. Am..

[9] Cupisti A., Brunori G., Di Iorio B.R., D’Alessandro C., Pasticci F., Cosola C., Bellizzi V., Bolasco P., Capitanini A., Fantuzzi A.L. (2018). Nutritional treatment of advanced CKD: Twenty consensus statements. J. Nephrol..

[10] Bajwa J., Munir U., Nori A., Williams B. (2021). Artificial intelligence in healthcare: Transforming the practice of medicine. Future Healthc. J..

[11] Aung Y.Y.M., Wong D.C.S., Ting D.S.W. (2021). The promise of artificial intelligence: A review of the opportunities and challenges of artificial intelligence in healthcare. Br. Med. Bull..

[12] Tai M.C. (2020). The impact of artificial intelligence on human society and bioethics. Tzu Chi Med. J..

[13] Chaudhuri S., Long A., Zhang H., Monaghan C., Larkin J.W., Kotanko P., Kalaskar S., Kooman J.P., Van Der Sande F.M., Maddux F.W. (2021). Artificial intelligence enabled applications in kidney disease. Semin. Dial..

[14] Yuan Q., Zhang H., Deng T., Tang S., Yuan X., Tang W., Xie Y., Ge H., Wang X., Zhou Q. (2020). Role of Artificial Intelligence in Kidney Disease. Int. J. Med. Sci..

[15] Davenport T., Kalakota R. (2019). The potential for artificial intelligence in healthcare. Future Healthc. J..

[16] Caldarini G., Jaf S., McGarry K. (2022). A Literature Survey of Recent Advances in Chatbots. Information.

[17] Li Y., Liang S., Zhu B., Liu X., Li J., Chen D., Qin J., Bressington D. (2023). Feasibility and effectiveness of artificial intelligence-driven conversational agents in healthcare interventions: A systematic review of randomized controlled trials. Int. J. Nurs. Stud..

[18] Gabarron E., Larbi D., Denecke K., Årsand E. (2020). What Do We Know About the Use of Chatbots for Public Health?. Stud. Health Technol. Inform..

[19] Suppadungsuk S., Thongprayoon C., Krisanapan P., Tangpanithandee S., Garcia Valencia O., Miao J., Mekraksakit P., Kashani K., Cheungpasitporn W. (2023). Examining the Validity of ChatGPT in Identifying Relevant Nephrology Literature: Findings and Implications. J. Clin. Med..

[20] Miao J., Thongprayoon C., Cheungpasitporn W. (2023). Assessing the Accuracy of ChatGPT on Core Questions in Glomerular Disease. Kidney Int. Rep..

[21] Potassium in Your CKD Diet. https://www.kidney.org/atoz/content/potassium-ckd-diet.

[22] Clase C.M., Carrero J.-J., Ellison D.H., Grams M.E., Hemmelgarn B.R., Jardine M.J., Kovesdy C.P., Kline G.A., Lindner G., Obrador G.T. (2020). Potassium homeostasis and management of dyskalemia in kidney diseases: Conclusions from a Kidney Disease: Improving Global Outcomes (KDIGO) Controversies Conference. Kidney Int..

[23] Holewinski T., Penniston K.L. (2018). Chronic Kidney Disease: Balancing Nutritional Needs with Nutrition Prevention of Kidney Stones. Nutrition Therapy for Urolithiasis.

[24] Phosphorus and Your Diet. https://www.kidney.org/atoz/content/phosphorus.

[25] Kovesdy C.P. (2022). Epidemiology of chronic kidney disease: An update 2022. Kidney Int. Suppl. (2011).

[26] Akhtar Z., Leung L.W., Kontogiannis C., Chung I., Bin Waleed K., Gallagher M.M. (2022). Arrhythmias in Chronic Kidney Disease. Eur. Cardiol. Rev..

[27] Brown S.J., Ruppe M.D., Tabatabai L.S. (2017). The Parathyroid Gland and Heart Disease. Methodist DeBakey Cardiovasc. J..

[28] Habas E., Eledrisi M., Khan F., Elzouki A.-N.Y. (2021). Secondary Hyperparathyroidism in Chronic Kidney Disease: Pathophysiology and Management. Cureus.

[29] León J.B., Sullivan C.M., Sehgal A.R. (2013). The Prevalence of Phosphorus-Containing Food Additives in Top-Selling Foods in Grocery Stores. J. Ren. Nutr..

[30] Ritz E., Hahn K., Ketteler M., Kuhlmann M.K., Mann J. (2012). Phosphate Additives in Food. Dtsch. Ärzteblatt Int..

[31] Hand D.J., Khan S. (2020). Validating and Verifying AI Systems. Patterns.

[32] Tsopra R., Fernandez X., Luchinat C., Alberghina L., Lehrach H., Vanoni M., Dreher F., Sezerman O.U., Cuggia M., De Tayrac M. (2021). A framework for validating AI in precision medicine: Considerations from the European ITFoC consortium. BMC Med. Inform. Decis. Mak..

[33] Grunhut J., Wyatt A.T., Marques O. (2021). Educating Future Physicians in Artificial Intelligence (AI): An Integrative Review and Proposed Changes. J. Med. Educ. Curric. Dev..

[34] Kalantar-Zadeh K., Moore L.W. (2020). Precision Nutrition and Personalized Diet Plan for Kidney Health and Kidney Disease Management. J. Ren. Nutr..

[35] Larivée N.L., Michaud J.B., More K.M., Wilson J.-A., Tennankore K.K. (2023). Hyperkalemia: Prevalence, Predictors and Emerging Treatments. Cardiol. Ther..

[36] Jovanovich A., Kendrick J. (2018). Personalized Management of Bone and Mineral Disorders and Precision Medicine in End-Stage Kidney Disease. Semin. Nephrol..

[37] He J., Baxter S.L., Xu J., Xu J., Zhou X., Zhang K. (2019). The practical implementation of artificial intelligence technologies in medicine. Nat. Med..

[38] Bohr A., Memarzadeh K. (2020). The rise of artificial intelligence in healthcare applications. Artificial Intelligence in Healthcare.

[39] Birkhäuer J., Gaab J., Kossowsky J., Hasler S., Krummenacher P., Werner C., Gerger H. (2017). Trust in the health care professional and health outcome: A meta-analysis. PLoS ONE.

[40] Charkviani M., Thongprayoon C., Tangpanithandee S., Krisanapan P., Miao J., Mao M.A., Cheungpasitporn W. (2022). Effects of Mediterranean Diet, DASH Diet, and Plant-Based Diet on Outcomes among End Stage Kidney Disease Patients: A Systematic Review and Meta-Analysis. Clin. Pract..

[41] Hansrivijit P., Oli S., Khanal R., Ghahramani N., Thongprayoon C., Cheungpasitporn W. (2020). Mediterranean diet and the risk of chronic kidney disease: A systematic review and meta-analysis. Nephrology (Carlton).

[42] Kim S.M., Jung J.Y. (2020). Nutritional management in patients with chronic kidney disease. Korean J. Intern. Med..

[43] Katirai A. (2023). The ethics of advancing artificial intelligence in healthcare: Analyzing ethical considerations for Japan's innovative AI hospital system. Front. Public Health.

[44] Arambula A.M., Bur A.M. (2020). Ethical Considerations in the Advent of Artificial Intelligence in Otolaryngology. Otolaryngol. Head Neck Surg..

[45] Garcia Valencia O.A., Suppadungsuk S., Thongprayoon C., Miao J., Tangpanithandee S., Craici I.M., Cheungpasitporn W. (2023). Ethical Implications of Chatbot Utilization in Nephrology. J. Pers. Med..

[46] Richardson J.P., Smith C., Curtis S., Watson S., Zhu X., Barry B., Sharp R.R. (2021). Patient apprehensions about the use of artificial intelligence in healthcare. Npj Digit. Med..

[47] Garcia Valencia O.A., Thongprayoon C., Jadlowiec C.C., Mao S.A., Miao J., Cheungpasitporn W. (2023). Enhancing Kidney Transplant Care through the Integration of Chatbot. Healthcare.

