# Supplementary material for: AI-Powered Renal Diet Support: Performance of ChatGPT, Bard AI, and Bing Chat
Source: Clin Pract. 2023 Sep 26;13(5):1160–72. doi: 10.3390/clinpract13050104 (PMC10605499; doi:10.3390/clinpract13050104)
Supplement: Supplementary file 1 [file clinpract-13-00104-s001.zip › clinpract-2604979-supplementary.pdf]

Supplementary Table S1 Potassium Identification Results by Chatbots

Diet	Potassium level	GPT 3.5 (1st test)	GPT 3.5 (2nd test)	GPT 4	Bard AI	Bing AI
Acorn squash	High	correct	Correct	correct	correct	correct
Apricots	High	correct	correct	correct	correct	correct
Artichoke	High	correct	incorrect	correct	correct	correct
Artichoke hearts	High	correct	incorrect	correct	correct	correct
Baked beans	High	correct	correct	correct	correct	correct
Baked potato	High	correct	correct	correct	correct	correct
Banana	High	correct	correct	correct	correct	correct
Beets	High	correct	correct	correct	correct	correct
Black beans	High	correct	correct	correct	correct	correct
Black-eyed peas	High	correct	correct	correct	correct	correct
Brussels sprouts	High	correct	correct	correct	correct	correct
Butternut squash	High	correct	correct	correct	correct	correct
Cantaloupe	High	correct	correct	correct	correct	correct
Carrot juice	High	incorrect	incorrect	correct	correct	correct
Chicory	High	correct	correct	correct	correct	correct
Chocolate milk	High	correct	correct	correct	correct	incorrect
Cocoa mix (made with milk)	High	incorrect	correct	correct	incorrect	incorrect
Cocoa mix (made with water)	High	incorrect	incorrect	correct	incorrect	incorrect
Collards	High	correct	correct	correct	correct	correct
Cowpeas	High	correct	correct	correct	correct	correct
Edamame	High	correct	correct	correct	correct	correct
Elderberries	High	correct	incorrect	correct	correct	correct
Enriched rice milk	High	correct	incorrect	correct	incorrect	incorrect
Enriched soymilk	High	correct	correct	correct	incorrect	incorrect
Fava beans	High	correct	correct	correct	correct	correct
Figs	High	correct	correct	correct	correct	correct
Firm tofu	High	correct	correct	correct	incorrect	incorrect
Flavored yogurt	High	correct	correct	correct	incorrect	incorrect
Gooseberries	High	correct	correct	correct	correct	correct
Green beans	High	correct	correct	correct	correct	correct
Guava	High	correct	correct	correct	correct	correct
Honeydew	High	correct	incorrect	correct	correct	correct
Hubbard squash	High	correct	correct	correct	correct	correct
pales or cactus leaves	High	correct	correct	correct	correct	correct
Kidney beans	High	correct	correct	correct	correct	correct
Kiwifruit	High	correct	correct	correct	correct	correct
Kohlrabi	High	correct	incorrect	correct	correct	correct

Lentils	High	correct	correct	correct	correct	correct
Lima beans	High	correct	correct	correct	correct	correct
low sodium V8 vegetable juice	High	incorrect	correct	incorrect	correct	correct
Mango	High	correct	correct	correct	correct	incorrect
Milk	High	incorrect	correct	correct	incorrect	correct
Mixed vegetables	High	correct	correct	correct	incorrect	correct
Mushrooms	High	correct	incorrect	correct	incorrect	incorrect
Navy beans	High	correct	correct	correct	correct	correct
Nectarine	High	correct	correct	correct	correct	correct
Orange	High	correct	correct	correct	correct	correct
Orange juice	High	correct	correct	correct	correct	correct
Papaya	High	correct	incorrect	correct	correct	correct
Parsnips	High	correct	correct	correct	correct	correct
Passion fruit juice	High	correct	correct	correct	incorrect	correct
Pinto beans	High	correct	correct	correct	correct	correct
Plain soymilk	High	correct	correct	correct	incorrect	incorrect
Plain yogurt	High	correct	correct	correct	incorrect	incorrect
Plantains	High	correct	correct	correct	correct	correct
Pomegranate	High	correct	correct	correct	correct	correct
Pomegranate juice	High	correct	correct	correct	correct	correct
Prickly pear	High	correct	correct	correct	correct	incorrect
Prune juice	High	correct	correct	correct	correct	correct
Prunes	High	correct	correct	correct	correct	correct
Pumpkin	High	correct	incorrect	correct	correct	correct
Refried beans	High	correct	correct	correct	correct	correct
Rutabaga	High	correct	correct	correct	correct	correct
Soybeans	High	correct	correct	correct	correct	correct
Spaghetti sauce	High	correct	correct	correct	correct	correct
Split peas	High	correct	correct	correct	correct	correct
Strawberries	High	incorrect	incorrect	correct	incorrect	incorrect
Sweet potatoes	High	correct	correct	correct	correct	correct
Sweet red cherries	High	correct	correct	correct	correct	incorrect
Swiss chard	High	correct	correct	correct	correct	correct
Tangerines	High	incorrect	incorrect	correct	incorrect	correct
Tomato	High	correct	incorrect	correct	correct	correct
Tomato juice	High	correct	correct	correct	correct	correct
Tomato paste	High	correct	correct	correct	correct	correct
Tomato sauce	High	correct	correct	correct	correct	correct
V8 vegetable juice	High	correct	correct	correct	correct	correct
Watermelon	High	incorrect	incorrect	correct	incorrect	incorrect

White beans	High	correct	correct	correct	correct	correct
Yam	High	correct	correct	correct	correct	correct
Yellow summer squash	High	correct	incorrect	correct	incorrect	correct
Zucchini	High	incorrect	incorrect	correct	incorrect	correct
Alfalfa sprouts	Low	incorrect	correct	correct	correct	correct
Apple	Low	correct	incorrect	correct	correct	correct
Apple cider	Low	incorrect	correct	correct	correct	correct
Apple juice	Low	incorrect	incorrect	correct	correct	correct
Applesauce	Low	incorrect	correct	correct	incorrect	correct
Apricot nectar	Low	incorrect	incorrect	correct	correct	correct
Asparagus	Low	incorrect	correct	correct	correct	correct
Avocado	Low	incorrect	incorrect	incorrect	incorrect	incorrect
Bean sprouts	Low	correct	correct	correct	correct	correct
Blackberries	Low	incorrect	incorrect	correct	correct	correct
Blueberries	Low	correct	correct	correct	correct	correct
Boysenberries	Low	incorrect	incorrect	correct	correct	correct
Butter head lettuce	Low	correct	correct	correct	correct	correct
Cabbage	Low	incorrect	correct	correct	correct	correct
Chickpeas	Low	incorrect	incorrect	incorrect	incorrect	correct
Coffee	Low	correct	correct	correct	correct	correct
Corn	Low	correct	correct	incorrect	correct	correct
Cranberries	Low	correct	correct	correct	correct	correct
Cranberry juice cocktail	Low	correct	correct	correct	incorrect	correct
Cranberry sauce	Low	incorrect	incorrect	correct	incorrect	correct
Cucumber	Low	correct	correct	correct	correct	correct
Dates	Low	incorrect	incorrect	incorrect	incorrect	correct
Eggplant	Low	incorrect	incorrect	correct	correct	correct
Fruit cocktail	Low	incorrect	correct	correct	correct	correct
Garbanzo beans	Low	incorrect	incorrect	incorrect	incorrect	correct
Grape juice	Low	incorrect	incorrect	correct	correct	correct
Grapefruit juice	Low	incorrect	incorrect	correct	incorrect	correct
Grapes	Low	incorrect	incorrect	correct	correct	correct
Iceberg lettuce	Low	correct	correct	correct	correct	correct
enriched rice milk	Low	correct	correct	incorrect	correct	incorrect
Jalapeno peppers	Low	incorrect	correct	incorrect	incorrect	correct
Japanese persimmon	Low	incorrect	incorrect	correct	incorrect	correct
Kale	Low	incorrect	incorrect	correct	correct	correct
Kumquats	Low	correct	correct	correct	correct	correct
Leek	Low	incorrect	incorrect	incorrect	correct	correct
Lemon	Low	correct	correct	correct	correct	correct

Lemon juice	Low	correct	correct	correct	correct	correct
Lemonade	Low	correct	correct	correct	correct	correct
Lime	Low	correct	correct	correct	correct	correct
Mandarin orange	Low	correct	correct	correct	correct	incorrect
Mango nectar	Low	incorrect	incorrect	correct	correct	correct
Okra	Low	incorrect	incorrect	incorrect	correct	correct
Onion	Low	correct	correct	incorrect	correct	correct
Papaya nectar	Low	incorrect	incorrect	correct	correct	incorrect
Parsley	Low	incorrect	correct	incorrect	correct	correct
Passion fruit	Low	incorrect	incorrect	incorrect	correct	incorrect
Pea pods	Low	correct	incorrect	incorrect	correct	incorrect
Peach nectar	Low	incorrect	incorrect	correct	correct	correct
Peanut butter	Low	incorrect	incorrect	incorrect	correct	correct
Pear nectar	Low	incorrect	incorrect	incorrect	correct	incorrect
Pears	Low	incorrect	incorrect	incorrect	correct	correct
Peas	Low	incorrect	incorrect	incorrect	correct	incorrect
Pineapple	Low	correct	incorrect	incorrect	correct	correct
Pineapple juice	Low	incorrect	incorrect	correct	correct	incorrect
Radishes	Low	correct	correct	incorrect	correct	incorrect
Raisins	Low	incorrect	incorrect	incorrect	incorrect	correct
Raspberries	Low	correct	correct	correct	correct	correct
Reduced calorie Cranberry juice cocktail	Low	correct	correct	correct	correct	correct
Romaine lettuce	Low	incorrect	correct	correct	correct	correct
Salsa	Low	correct	correct	incorrect	correct	correct
Serra peppers	Low	incorrect	incorrect	incorrect	incorrect	incorrect
Snow peas	Low	incorrect	correct	incorrect	correct	incorrect
Soft tofu	Low	incorrect	incorrect	incorrect	correct	correct
Sour cherries	Low	incorrect	incorrect	correct	incorrect	correct
Spaghetti squash	Low	correct	correct	incorrect	correct	incorrect
Sweet cherries	Low	incorrect	incorrect	incorrect	incorrect	correct
Water chestnuts	Low	correct	correct	incorrect	correct	incorrect
White mushrooms	Low	incorrect	correct	correct	correct	correct

Supplementary Table S2 Accuracy of AI Models in Identifying High Phosphorus Foods

Diet	Phosphorus level	GPT 3.5 (1st test)	GPT 3.5 (2nd test)	GPT4	Bard	Bing
Blue cheese	High	correct	correct	correct	correct	correct
Cheddar cheese	High	correct	correct	correct	correct	correct
Colby cheese	High	correct	correct	correct	correct	correct
Mozzarella cheese	High	correct	correct	correct	correct	correct
Swiss cheese	High	correct	correct	correct	correct	correct
Provolone	High	correct	correct	correct	correct	correct
Ricotta	High	correct	incorrect	correct	correct	correct
Cheese spread	High	correct	incorrect	correct	correct	correct
Cream	High	correct	correct	correct	correct	correct
Half and half	High	correct	correct	correct	correct	correct
Evaporated milk	High	correct	correct	correct	correct	correct
Condensed milk	High	correct	correct	correct	correct	correct
Ice cream	High	correct	correct	correct	correct	correct
Frozen yogurt	High	correct	correct	correct	correct	correct
Plain cow milk	High	correct	correct	correct	correct	correct
Buttermilk	High	correct	correct	correct	correct	correct
Pudding	High	correct	correct	correct	correct	correct
Custard	High	correct	correct	correct	correct	correct
Soy milk	High	correct	correct	correct	correct	correct
Yogurt	High	correct	correct	correct	correct	correct
Kidney beans	High	correct	correct	correct	correct	correct
Black beans	High	correct	correct	correct	correct	correct
Pinto beans	High	correct	correct	correct	correct	correct
Garbanzo beans	High	correct	correct	correct	correct	correct
Navy beans	High	correct	correct	correct	correct	correct
Walleye fish	High	correct	correct	correct	correct	correct
Canned sardines fish	High	correct	correct	correct	correct	correct
Hummus	High	correct	correct	correct	correct	incorrect
Lentils	High	correct	correct	correct	correct	incorrect
Black-eyed peas	High	correct	correct	correct	correct	correct
Split green peas	High	correct	correct	correct	correct	correct
Ham	High	correct	correct	correct	correct	correct
Liver	High	correct	correct	correct	correct	correct
Processed meats	High	correct	correct	correct	correct	correct
Nuts	High	correct	correct	correct	correct	correct
Seeds	High	correct	correct	correct	correct	correct

Peanut butter	High	correct	correct	correct	correct	correct
Soy burgers	High	correct	correct	correct	correct	correct
Bean-based veggie burgers	High	correct	correct	correct	correct	correct
Firm Tofu	High	correct	correct	correct	correct	incorrect
Extra-firm Tofu	High	correct	correct	correct	correct	correct
Corn	High	correct	correct	correct	correct	correct
Peas	High	correct	correct	correct	correct	correct
Beer	High	correct	incorrect	correct	correct	correct
Carbonated colas	High	correct	correct	correct	correct	correct
Pepper-type soft drink	High	correct	correct	correct	correct	correct
Fruit-falvored soft drinks	High	correct	correct	correct	correct	correct
Bran flakes	High	correct	correct	correct	correct	correct
Bran buds	High	correct	correct	correct	correct	correct
Bran muffin	High	correct	correct	correct	correct	correct
Biscuits	High	correct	incorrect	correct	correct	correct
Pancakes	High	correct	correct	correct	correct	correct
Waffles	High	correct	correct	correct	correct	correct
Frosted Mini Wheats	High	correct	correct	correct	correct	correct
Granola	High	correct	correct	correct	correct	correct
Millet	High	correct	incorrect	correct	correct	correct
Quinoa	High	correct	incorrect	correct	correct	correct
Raisin Bran	High	correct	correct	correct	correct	correct
Oat bran	High	correct	correct	correct	correct	correct
Old-fashioned oatmeal	High	correct	correct	correct	correct	correct
Wheat germ	High	correct	correct	correct	correct	correct
Wheat bran	High	correct	correct	correct	correct	correct
Whole-grain crackers	High	correct	correct	correct	correct	correct
Whole-wheat breads	High	correct	correct	correct	correct	correct
Whole-grain breads	High	correct	correct	correct	correct	correct
Wild rice	High	correct	correct	correct	correct	correct
Chocolate	High	correct	correct	correct	correct	correct
Doughnut	High	correct	correct	correct	correct	correct
Cinnamon roll	High	correct	correct	correct	correct	correct
Danish	High	correct	correct	correct	correct	correct
éclair	High	correct	correct	incorrect	correct	correct
Chocolate cream pie	High	correct	correct	incorrect	correct	correct
Coconut cream pie	High	correct	correct	incorrect	correct	correct
Custard pie	High	correct	correct	incorrect	correct	correct
Pecan pie	High	correct	correct	incorrect	correct	correct
Pumpkin pie	High	correct	correct	incorrect	correct	correct

Chocolate toaster pastry	High	correct	correct	incorrect	correct	correct
Fat-free cream cheese	High	incorrect	incorrect	incorrect	correct	correct
Almond milk	High	incorrect	incorrect	incorrect	correct	correct
Pollock fish	High	incorrect	incorrect	incorrect	correct	correct
Avocado	High	incorrect	incorrect	incorrect	correct	correct
Mushrooms	High	incorrect	incorrect	incorrect	correct	correct
Coconut	High	incorrect	incorrect	incorrect	correct	correct
Brown rice	High	incorrect	correct	incorrect	correct	correct
Regular Cheerios	High	incorrect	incorrect	incorrect	correct	incorrect
Honey Nut Cheerios	High	incorrect	incorrect	incorrect	correct	incorrect
Corn muffin	High	incorrect	correct	incorrect	correct	incorrect
Corn tortilla	High	incorrect	incorrect	incorrect	correct	incorrect
Instant oatmeal	High	incorrect	correct	incorrect	correct	incorrect
Caramels	High	incorrect	incorrect	incorrect	correct	incorrect
Sugar-free gelatin	High	incorrect	incorrect	incorrect	correct	incorrect
